# Microphase Segregation of Diblock Copolymers Studied by the Self-Consistent Field Theory of Scheutjens and Fleer

**DOI:** 10.3390/polym10010078

**Published:** 2018-01-17

**Authors:** Merve Mocan, Marleen Kamperman, Frans A. M. Leermakers

**Affiliations:** Physical Chemistry and Soft Matter, Wageningen University, Stippeneng 4, 6708 WE Wageningen, The Netherlands; merve_mocan@yahoo.com (M.M.); marleen.kamperman@wur.nl (M.K.)

**Keywords:** microphase segregation, self-consistent field theory, block copolymers

## Abstract

We used the self-consistent field (SCF) formalism of Scheutjens and Fleer (SF-SCF) to complement existing theoretical investigations on the phase behavior of block copolymer melts. This method employs the freely jointed chain (FJC) model for finite chain length and systematic differences exist compared to the classical SCF predictions. We focus on the critical and hexagonal (HEX) to lamellar (LAM) phase transition region at intermediate and strong segregations. Chain length (*N*) dependence of the critical point ($\chi^{cr}$) was found to be $\chi^{cr}N=10.495\left(1+4/N\right)$. The characteristic spacing (*D*) of LAM was found as $D=4/3\sqrt{N}$ at the critical conditions. We present SF-SCF predictions for the phases single gyroid (SG), double gyroid (DG) and hexagonally perforated lamellar (HPL), in the region where HEX and LAM compete. At χN=30, N=300; we found SG and HPL were metastable with respect to LAM or HEX, DG was stable in a narrow region of the asymmetry ratio. In contrast to the latest predictions, at strong segregation χN=120, DG was found to be metastable. From the structural evolution of HPL, we speculate that this may be an intermediate phase that allows the system to go through various connectivity regimes between minority and majority blocks.

## 1. Introduction

Typically, when two chemically different polymers with a small but positive interaction parameter χ are mixed, when time permitted, they will demix into two macroscopic phases of which one is rich in one polymer and depleted in the other while the other phase obtains the opposite composition [1]. The phase diagram is characterized by a critical volume fraction and a critical interaction parameter. When the two polymers are equally long (each composed of *N* segments) the critical volume fraction ($\varphi^{cr}$) is, for symmetry reasons, $\varphi^{cr}=0.5$, while the critical interaction parameter ($\chi^{cr}$) decreases with the chain length (*N*) as $\chi^{cr}=2/N$. In the critical region, one finds power-law behavior of the interfacial tension (γ) $\gamma \propto {\left(\Delta \chi \right)}^{\alpha }={\left(\chi -\chi^{cr}\right)}^{\alpha }$, the density difference between the phases (Δφ) $\Delta \varphi \propto {\left(\Delta \chi \right)}^{\beta }$ and width of the interface $W={\left(\Delta \chi \right)}^{\delta }$. The first two tend to go to zero (both α and β are larger than zero), whereas the latter quantity diverges (δ<0), upon an approach towards the critical point. In the mean field theory, α=3/2, β=1/2, and δ=−1/2 [2,3].

When the two polymers are combined into a block copolymer, the tendency to demix is still present, but macrophase segregation is impossible. Instead, microphase segregation occurs. There are domains rich in one segment type and depleted in the other, while the other domains develop with opposite composition. Again, a phase diagram may be found which separates a homogeneous from a microphase segregated state. The lowest possible interaction parameter for which segregation is possible, occurs for the case that the two blocks are equally long (f=0.5, where *f* is the asymmetry ratio between the blocks), and again the critical interaction parameter decreases with overall chain length roughly as $\chi^{cr}=10.5/N$. In the critical region, we again should expect power-law dependences, e.g., for the free energy density (*g*) (compared to the free energy of the homogeneous phases) $-g\propto {\left(\Delta \chi \right)}^{\alpha }$, and the difference of volume fractions in the two regions $\Delta \varphi \propto {\left(\Delta \chi \right)}^{\beta }$ with both α and β are positive. Below, we will show that in this case, α=2 and β=1/2. However, as there is a finite characteristic length scale in the system (the dimension of the microphases), the width of the interface cannot diverge because it is upper bounded by this characteristic length scale.

Here, we use a self-consistent field (SCF) theory with the discretization scheme of Scheutjens and Fleer (SF-SCF) to study microphase segregation [4,5]. Apart from the use of a lattice, there are noticeable differences between the classical SCF models used in the literature and SF-SCF. To be more specific, SF-SCF implements not only local but also non-local contributions to the contact interactions in the segment potentials (see below and the Appendix for more details), whereas, in the literature, the non-local effects tend to be neglected for the study of microphase segregation [6,7,8]. This is a minor issue when the gradients in the densities are very small, e.g., close to the critical point, but it becomes more important when the gradients in densities are large, e.g., for strong segregation. The non-local effects are also thought to be more relevant for finite chain lengths and less important for the infinite chain length limit. Typically, within SF-SCF, the focus is explicitly on finite chain lengths. The infinite chain length limit is only found by extrapolation. SF-SCF has been used successfully for modeling inhomogeneous polymer and surfactant systems, including self-assembly of surfactants and lipids in aqueous solutions [9,10,11,12,13,14]. Recently, some Hessian-free search methods were introduced in SF-SCF, which now allows us to consider three-gradient problems and hence to consider microphase segregation of block copolymers. The target of this paper is to apply SF-SCF to microphase segregation and where possible compare to data from literature. For this reason, it is natural to first focus on the critical region before addressing finite chain length effects in other parts of the phase diagram such as for the lamellar-to-hexagonal phase boundary.

In a recent paper, Matsen employed a freely jointed chain SCF (FJC-SCF) approach for microphase segregation [8]. In this paper, polymers that have rather short chain length were used from which it was already clear that there are significant finite chain length effects. We haste to mention that this FJC-SCF approach still differs from SF-SCF because, in the latter, non-local contributions for counting the interactions are included. We argue that this effect may be noticed when the interface between the two domains is relatively sharp. Indeed, below, we report noticeable differences between FJC-SCF and SF-SCF and attribute these to the different implementations of the otherwise similar SCF equations on a lattice model.

Unlike in the macrophase segregation for which $\chi^{cr}=2/N$, for microphase segregation, we expect that the quoted value $\chi^{cr}=10.495/N$ is not the exact (mean field) result for finite chain lengths [6,15,16,17,18,19]. More specifically, FJC-SCF [8] results already report noticeable changes of the critical point for finite chain lengths. We also find such deviations, but our results differ noticeably from the reported effect of Matsen [8]. In the literature, there are few other reports about possible corrections for the critical point deviating from χN=10.495 due to fluctuations (beyond mean field) [17]. It must be clear, however, that our finite chain length corrections that are discussed below are not related to the fluctuations discussed by Fredrickson [17], but rather of the same type as reported by Matsen [8].

In practice, block copolymers are rarely found in the weak segregation limit. When interactions are stronger, the phase diagram progressively becomes richer. One can find various topologies of microstructures that result from a balance of opposite tendencies: (i) the larger the interaction parameter χ, the higher the energetic cost to have an A–B interface in the system and therefore the tendency to reduce this area becomes progressively stronger; and (ii) a small interfacial area per molecule will imply the brush-like stretching of the two blocks to avoid overcrowding effects. Of course, this implies a conformational entropy loss. Hence, the system will try to minimize stretching and this will increase the area per molecule; (iii) the copolymer will need to occupy the total volume and leave no voids. Therefore, the stretching of the chains cannot always be homogeneous: there may be regions in the volume that are further away from the ‘interface’ than others, and the filling of these distant volumes require extra stretching of chain parts. Inhomogeneous stretching is avoided as much as possible.

These above set of tendencies are not easily accounted for analytically and that is why microphase segregation is the domain of numerical analysis. It is well known that the outcome depends on the fraction $f=f_{A}=N_{A}/\left(N_{A}+N_{B}\right)$ in an $A_{N_{A}}B_{N_{B}}$ block copolymer. At extreme values of *f*, we have spherical domains of the minority phase and these domains may be packed in regular order. At intermediate values, we have a cylindrical topology and these are packed in a hexagonal packing, and, around f=1/2, there is a lamellar phase. Depending on the strength of the interactions, other competing mesostructures are found between the hexagonal (HEX) and lamellar (LAM) phases. The most prominent examples are (double) gyroid, double diamond (DD) and hexagonally perforated lamellae (HPL).

Single gyroid (SG) (space group $I4_{1}32$) was observed for the first time in 1967 by Luzatti et al. in strontium soap surfactants [20] and identified by Alan Schoen who classified 17 such minimal surfaces, named it as gyroid or Schoen G surface [21]. However, now, the term gyroid is being used more commonly for double gyroid structures. In nature, SG structure was detected in butterfly wing scales. The formation of these biophotonic nanostructures, which produce the brilliant colors on butterfly wings, was explained as the deposition and polymerization of chitin on the initially formed double gyroid (DG) structure and, as the cell dies, air replaces cell content and an SG structure consisting of chitin and air remains [22]. Similar SG structures were also found in the retinal cone mitochondria of tree shrews [23]. SG structure was rarely observed compared to DG and DD. Even though SG was not found in diblock copolymers, a current approach is to produce SG templates from a DG forming ABC block copolymers such as poly(isoprene-*b*-styrene-*b*-ethylene oxide) by etching the blocks and metal deposition [24]. The double gyroid (space group $Ia\bar{3}d$), which consists of three continuous subvolumes with two non-intersecting SG’s with the same volume and the remaining volume as the matrix phase, was discovered in 1986 by first being misinterpreted as DD [25], and then correctly identified by two independent groups in 1994 [26,27]. DG was identified in various diblock copolymers [28,29], ABA triblocks [30,31] as well as ABC triblock copolymers [32]. In the SCF calculations of Matsen et al., the HPL (space group $R\bar{3}m$) phase was predicted to be nearly stable for diblock copolymers [6]. Wang et al. speculated that HPL is an intermediate state during HEX-DG transition in diblock copolymers [33]. Nevertheless, the metastable HPL phase could be experimentally obtained in diblock copolymers [34,35]. Another common mesophase, the double diamond (space group $Pn\bar{3}m$), was first discovered in styrene-isoprene star block copolymers [25]. The DD phase was also identified for diblock copolymers [36] and their homopolymer blends [37]. After a re-examining of some of the obtained DD morphologies, it was figured out that they only misidentified DG morphologies, since DG highly resembled DD in TEM images [38]. We present a set of results for the HPL case for various values of *f* for given χN. We decide to show some snapshot of the density distributions in the unit cells for the phase to highlight how such a phase can change its appearance. We do this because these profiles are ’inspiring’, illustrated the rich physics of microphase segregation, and because such profiles are rarely found in the literature.

Regarding the stability of DG, there remain points of debate. More specifically, according to the early SCF calculations performed by Matsen and Bates [6], the DG cannot be stable at strong segregation, and it was found that, beyond the triple point around χN=60 [39], the DG loses from HEX or LAM. More recent predictions of Cochran et al. claim that DG is stable even beyond χN=100 with a broadening stability window [7]. The stability question of DG phase for finite chain length still await definite answers. As mentioned above, for finite chain length and strong segregation, it may be necessary to have a chain model that remains accurate when the chains become strongly stretched. The freely jointed chain (FJC) is expected to behave better than the Gaussian chain in this respect. Also for strong segregation, the interfaces become more narrow and the gradients in density increase. In such a situation, a theory that accounts for non-local effects in the segment potential is expected to be more accurate than theories that ignore these subtleties. It is therefore of interest to use the SF-SCF method for the modeling of microphase segregation in this limit.

Below, we consider the phase boundary between HEX and LAM phases by taking the SG, DG, DD and HPL phases into account. Using the SF-SCF approach, we confirm the literature prediction that there exists a narrow region in *f* for which at χN=30 and N=300 diblock copolymers prefer the DG phase over the HEX or LAM phases. Other phases such as the DD, SG and HPL phases were found to be metastable. In contrast to the more recent predictions, we confirm early results of Matsen and Bates [6,39] by giving evidence that, at strong segregation (χN=120), the HEX phase gives directly way to the LAM phase as now also the DG is metastable.

The remainder of this paper is the following. We will first present aspects relevant for the modeling of microphase segregation of the SF-SCF approach. The idea is to give sufficient information to the reader to understand how the results were generated. Other details of the modeling are deferred to the Appendix. In the results section, we will first outline the procedures that were followed to identify the relevant results of the SF-SCF modeling. We will subsequently present our result for the near critical region of the phase diagram and then proceed with the systematic analysis of the HEX to LAM phase transition regions at intermediate and strong segregation regimes. In the discussion, we will elaborate on our expectations about how the SF-SCF method can be further used for the analysis of block copolymer self-assembly. At the end, we formulate our conclusions.

### SF-SCF Characteristics and Parameters

The SCF machinery follows from optimizing a mean field free energy functional that is expressed in terms of segment volume fraction profiles and complementary segment potential profiles (see Appendix A). Both the potentials and the segment densities are a function of the spatial coordinates. The rule about how to compute the potentials from the segment densities follows from the optimization of the free energy with respect of the segment densities. The rule about how to compute the segments densities from the segment potentials follows from the minimization of the free energy with respect to the segment potentials. When we follow both rules and implement the incompressibility conditions, we can evaluate the equilibrated free energy (and obtain relevant thermodynamics from this) and evaluate the relaxed structural details of the system. The input for this machinery consists of four elements: (i) info on the molecular components. In the current system, there is only one type of molecule, namely a block copolymer $A_{N_{A}}B_{N_{B}}$ in the system, where $N_{A}$ is the degree of polymerization of the block A and $N_{B}$ is the degree of polymerization of block B. The molecules follow the FJC model for their conformational degrees of freedom. This chain model implements a finite chain extension, as all chain bonds are fixed in length. It ignores bond angle correlations. This implies that the statistical segments can go in any direction on the lattice including back folding. The latter obviously is an approximation, but we note that this excluded volume error is partially corrected by the compressibility condition; (ii) info about the interactions. As we have no solvent, there is only one relevant interaction parameter $\chi =\chi_{AB}$. We thus expect that segments only feel each other when they occupy nearest neighbor sites. The number of segment–segment contacts is estimated using the well-known Bragg–Williams (mean field) approximation; (iii) a specification of the coordinate system. Mostly, we consider an elementary 3-gradient (*x*-*y*-*z*) cell, which can be used to construct the complete spatial distribution of the copolymers. For the HEX phase, we can reduce the calculations to two gradients (*x*-*y*) while the cylinders lie in the *z*-direction, and, for the LAM phase, we consider only the direction normal to the planes (*x*-direction), which allows a reduction to a one-gradient calculation; and (iv) specification of the boundary conditions. In all the three-gradient cases we have implemented periodic boundaries, in the other phases we have used reflecting (mirror-like) boundaries. Below, we will pay attention to both elements (iii) and (iv) for each type of calculation that is discussed.

It is important to mention the four most important differences of the SF-SCF method compared to the SCF methods typically used for microphase segregation: (A) the SF-SCF method needs an initial guess that is then iteratively adjusted using a Hessian-free optimization method (for example, a steepest decent (SD) or a direct inversion in the iterative subspace (DIIS) scheme) [40,41]. This method does not need any further input on the symmetry of the solution and the precision does not depend on e.g., the specification of test-functions of any sort; (B) we have implemented the FJC model. Within this model, there exists an efficient propagator formalism to compute the volume fractions (see Appendix A). The chain model is appropriate for finite chain lengths and is expected to outperform the Gaussian chain model as soon as the chains become strongly stretched. This is more of an issue at strong segregation than at weak segregation; (C) the length scale of the segments (bond length) is also used to discretize space (lattice model). Other numerical SCF methods also rely on a discretization scheme, but, in SF-SCF, the discretization is built in more rigidly than in alternative approaches; (D) the SF-SCF model features segment potentials u(r) that include non-local contributions. The physical meaning of the segment potential is the work of bringing a segment from the reference phase (where the potential is zero) to the coordinate *r*. Apart from a contribution that is adjusted to obey to the incompressibility relation, there is a contribution due to the segment interactions, i.e., $E_{X}\left(r\right)$ for segment type *X* at coordinate *r*. As specified in the Appendix, this contribution implements the Bragg–Williams approximation and typically is given for segment A by $E_{A}\left(r\right)=\chi \varphi_{B}\left(r\right)$ and a similar equation applies for segment type B. This local definition of the interactions is correct/accurate in the absence or vanishing gradients in the segment density. In SF-SCF, one typically accounts for the gradients and the interactions are computed by $E_{A}\left(r\right)=\chi \langle \varphi_{B}\left(r\right)\rangle =\chi \varphi_{B}\left(r\right)+1/6\left(\partial^{2}\varphi_{B}\left(r\right)/\partial r^{2}\right)$ (see Appendix A). The latter contribution implements information on the ‘curvature’ of the density profile. Similar ‘corrections’ are implemented in the free energy functional. The curvature correction in the segment potential is referred to as the non-local contribution because, in the lattice, the second derivative is implemented by using the local averaging of the segment densities around a specified coordinate (see Appendix A).

In the following, we will first discuss how relevant SF-SCF results are obtained. Typical results for various mesophases are discussed and evaluated. This discussion is followed by a systematic analysis of the systems near the critical region. After this, we report on the spacing of the LAM phase for systems that are not near the critical point. The remainder of the results is focused on the transition region between the HEX and LAM phase and more specifically to the question of (meta)stability of the DG phase.

## 2. Results

### 2.1. Box Size Adjustment for Free Energy Optimization

As explained above (and in the Appendix), the SCF free energy features segment potentials and segment densities that are mutually dependent. The optimization of it leads to rules of which the fixed point is known as the SCF solution. Let us now assume that we have such an SCF solution, and we can evaluate the free energy using Equation (A4). One can easily see that, for an SCF solution, Equation (A4) can be simplified resulting in:

(1) $$G\equiv G-G^{b}=-n\ln\frac{q}{V}-\sum_{r}\alpha \left(r\right),$$

where the free energy of the homogeneous reference (the bulk) as the reference as usual, *q* is the single chain partition function, which can be computed with the propagator formalism from the segment potentials, *V* the system volume and α(r) is the value of the Lagrange field that takes values such that the system is incompressible at each coordinate, i.e., $\varphi_{A}\left(r\right)+\varphi_{B}\left(r\right)=1$. To compare systems with different size and structure to each other, it is appropriate to evaluate the free energy density for each of these systems:

(2) $$g=\frac{G}{V}.$$

Typically, the free energy density is negative because the microphase segregated state develops spontaneously from the homogeneous state. The system with the lowest free energy is the preferred one.

**Figure 1:**
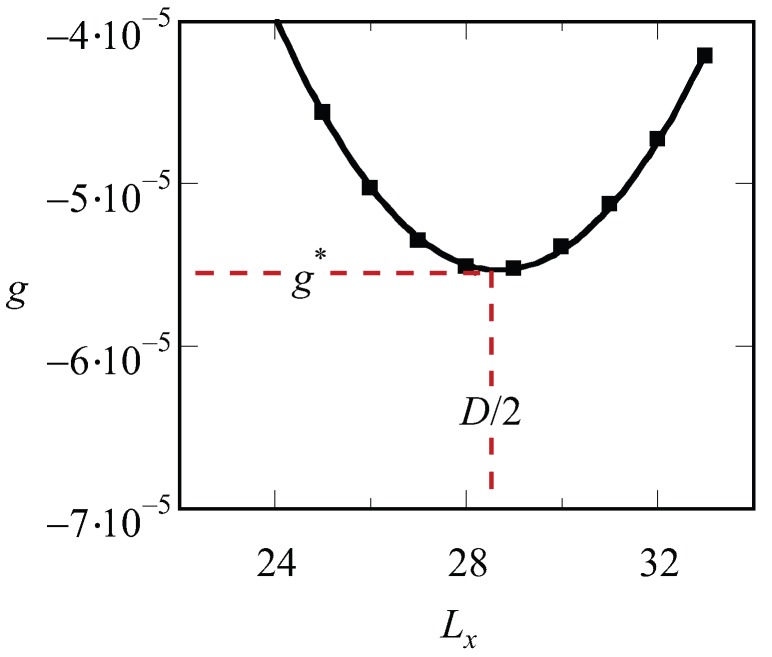
Free energy density *g* (in units of $k_{B}T/b^{3}$) as a function of the spacing $L_{x}$ for a LAM phase f=0.5, N=1600 and χ=0.9. The line is a parabolic fit through the data points. The optimal spacing *D* (in units *b*) as well as the corresponding optimal free energy density are indicated.

Inspection of the procedure reveals that we should fix the values of $L_{x}$, $L_{y}$ and $L_{z}$ before we can start solving the SCF equations. Hence, we end up with a free energy density that is a function of the specified spacing *D*, i.e., g=g(D). There is no guarantee that, for an arbitrary choice of the system size, the optimized SCF free energy density is at its minimal value. Therefore, we need to vary the value of $L_{x}$ in the lamellar phase, or the combination of $L_{x}$ and $L_{y}$ in the hexagonal phase or the value of $L=L_{x}=L_{y}=L_{z}$ in (e.g.) the gyroid case to find the optimal spacing $D=L_{x}^{*}$. A typical result of such procedure for a lamellar phase is presented in Figure 1. The free energy that is optimized with respect to the spacing is labeled by an asterisk, $g^{*}$.

Apart from the system size we need, though the choice of the number of gradient directions and the type of boundary condition, to specify the phase of choice of considerations. To illustrate this, we will visit the topologies that are used below, namely the LAM, HEX, SG, DG, DD and HPL phases in order.

### 2.2. The Lamellar Phase

For a LAM phase, we reduce the calculations to the one-gradient case r=x and the mean field approximation is applied in *y*- and *z*-directions. The system size is given by $L_{x}$ and we use reflecting boundary conditions:

(3) φ(0)=φ(1),

(4) $$\varphi \left(L_{x}+1\right)=\varphi \left(L_{x}\right).$$

All other quantities that are a function of the spatial coordinate, such as the segment potentials, the end-point distributions follow these rules. In this case, the free energy *G* is per unit area and the free energy density is found by $g=G/L_{x}$. Typically, we will have one A–B interface somewhere half-way in the ‘box’ (depending on *f*) and the lamellar spacing is given by $D=2L_{x}$. We select the A-rich domain to be at low *x*-values and the B-rich domain at high *x*-values by means of an initial guess for the segment potentials.

**Figure 2:**
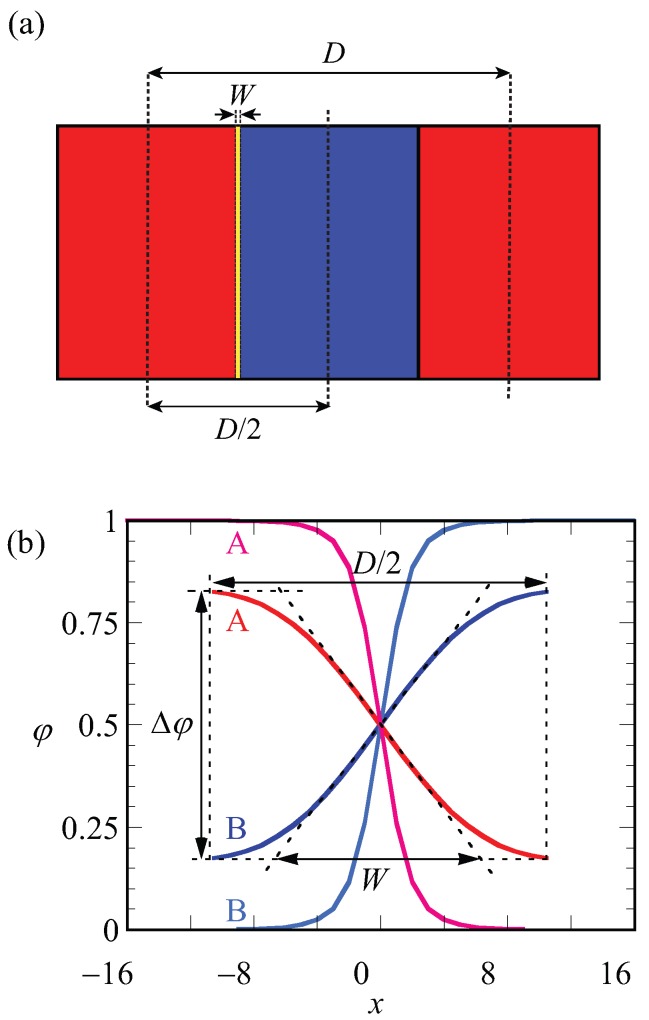
(**a**) Schematic representation of symmetrical LAM phase. Red and blue regions indicate blocks of A and B, respectively. The lamellar spacing *D* and the width of the interface *W* are indicated; (**b**) SF-SCF density profiles (φ) as a function of layer number *x* where x=0 is taken halfway the A–B interface. Parameters: f=0.5 and N=1000; red and blue lines are for weak segregation χN=12, optimal spacing is D/2=21 of A and B blocks, respectively, and pink and light blue lines are for strong segregation χN=75, D/2=31 of A and B blocks, respectively. The parameters Δφ, *D* and *W* are stated for χN=12.

A schematic representation of the LAM phase and the optimized (with respect to the spacing) density profile are given in Figure 2a,b. In Figure 2a, red and blue regions belong to the A and B blocks, respectively. The spacing (*D*) and width of the interface (*W*) are indicated on the lamellae. The same parameters (*D* and *W*) are defined more precisely on the density profiles $\varphi_{A}\left(x\right)$ and $\varphi_{B}\left(x\right)$ shown in Figure 2b. In this graph, the density difference $\Delta \varphi =\varphi_{A}-\varphi_{B}=1-2\varphi_{B}$, which φ is also the volume fraction midway in the A-rich phase, is indicated. As the coordinate x=0 is fixed to the position of the steepest gradients in the density, the evaluation of the density difference takes place at $x=-\frac{D}{4}$: $\phi =\varphi (-\frac{D}{4})$. Finally, the width of the interface, as graphically illustrated in Figure 2, is computed from the profiles according to

(5) $$W=\frac{\Delta \varphi }{\varphi_{A}\left(-\frac{1}{2}\right)-\varphi_{A}\left(\frac{1}{2}\right)}.$$

In Figure 2b, the curves in black belong to the density profiles of A and B blocks for χN=12 in the weak segregation regime where the optimized spacing is D=42, whereas density profiles shown in light gray belong to the A and B blocks of a copolymer in the strong segregation region of χN=75 with optimized spacing D=64. For weakly segregated block copolymers, Δφ easily deviates highly from unity and its value approaches zero in the limit towards the critical point, whereas it approaches unity for strong segregation limits. From its definition (Equation (5)), it is easily seen that the width of the interface *W* is small for strong segregation and increases upon the approach towards weak segregation. Obviously, *W* cannot exceed D/2.

### 2.3. The Hexagonal Phase

The hexagonal packing of cylindrical domains of the minority phase surrounded by the majority phase requires a two-gradient computation box. Ideally, the ratio of the box sizes in the gradient directions is $L_{x}/L_{y}=\sqrt{3}$. Below, we will accept small errors in the lattice dimensions and consider integer values for the system size in the *x*-gradient direction, $L_{x}$, and *y*-direction, $L_{y}$, while we apply a mean field approximation in the *z*-direction. For a given SCF fixed point, the free energy of *G* is given per unit area and the free energy density follows from $g=G/L_{x}L_{y}$. The lattice spacing is given by $D=\sqrt{L_{x}^{2}+L_{y}^{2}}$. Hence, when the correct ratio is implemented, we have $D=2L_{y}$. We follow the same definition for the spacing *D* when integer values are used for the sizes of the system in the gradient directions. While optimizing the lattice spacing, we adjust the combinations of $L_{x}$ and $L_{y}$ such that the $\sqrt{3}$ ratio is met as closely as possible (excluding the settings for which the ratio is far from the ideal value). However, there is always some imprecision remaining on the spacing values obtained for this phase and therefore we typically slightly overestimate the free energy. As in the LAM case, for the HEX phase, reflecting boundary conditions are implemented. That is, both in the *x*- and *y*-directions, the densities as well as the potentials and thus the end-point distributions obey the implementation of zero gradients across the boundaries as given in Equations (3) and (4).

**Figure 3:**
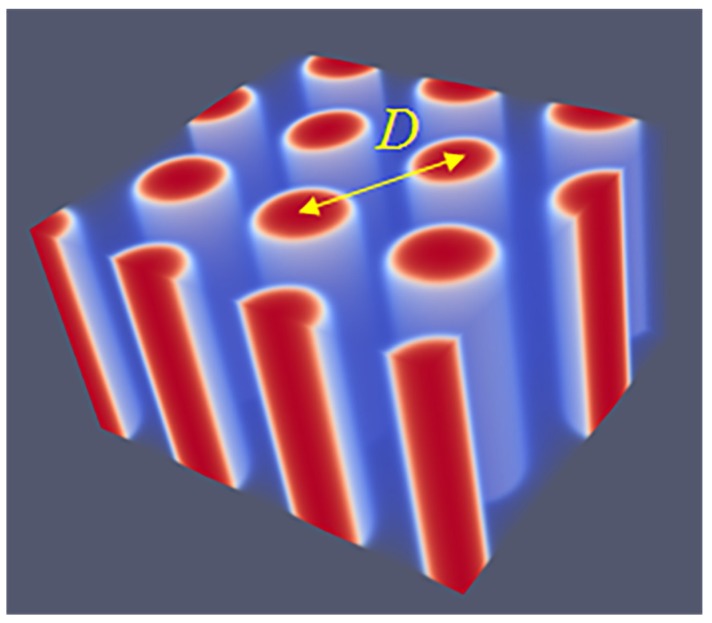
SF-SCF predictions for the density profile of HEX phase for D=60, N=1000, χN=30 and f=0.30. The spacing *D* is indicated. Color coding is as follows. Regions rich in the minority block (segment type A) are given in red, and the blue regions are rich in the majority phase (segment type B). The majority phase (blue) is made more transparent the higher its density is for presentation purposes. Mirror-like boundary conditions apply in all directions.

In Figure 3, a typical result for the density profile of HEX phase is given for D=60, which happens to be close to the optimal spacing for N=1000, χN=30 and f=0.30. The two-gradient results are ‘multiplied’ 8 times in *x*- and *y*-directions and about 50 times in the *z*-directions. Here and below, we give equal density color maps wherein the minority phase is given in red, while the majority phase is in blue (more intense color implies a higher density; the in between white color is the interface). In addition, the blue domain is made ‘transparent’ to allow the visualization of the cylinders in the HEX phase more clearly. It is possible to give, similarly as in the LAM phase a cross-sectional density profile. We do not show such a profile because the features (apart from the fact that the interface is not centered) are quite similar to those given in Figure 2b.

### 2.4. Various Mesophases That Require Three-Gradient SF-SCF

There are many three-gradient solutions of the SCF equations. Some of these, e.g., the Im3m cubic phase, require reflecting boundary conditions [13]. Here, our interest is in, e.g., SG and DG structures that lack symmetry planes. For such structures, one requires periodic boundary conditions. We restrict our analysis to three-gradient SF-SCF computations with equal sizes in the three gradient directions: $L\equiv L_{x}=L_{y}=L_{z}$. The periodic boundaries are implemented by realizing that coordinates 1 and *L* are ‘neighbors’ where it is understood that the potentials and end-point distributions follow the same rules:

(6) $$\begin{array}{c} \varphi \left(0,y,z\right)=\varphi \left(L_{x},y,z\right),\\ \varphi \left(L_{x}+1,y,z\right)=\varphi \left(1,y,z\right),\\ \varphi \left(x,0,z\right)=\varphi \left(x,L_{y},z\right),\\ \varphi \left(x,L_{y}+1,z\right)=\varphi \left(x,1,z\right),\\ \varphi \left(x,y,0\right)=\varphi \left(x,y,L_{z}\right),\\ \varphi \left(x,y,L_{z}+1\right)=\varphi \left(x,y,1\right). \end{array}$$

**Figure 4:**
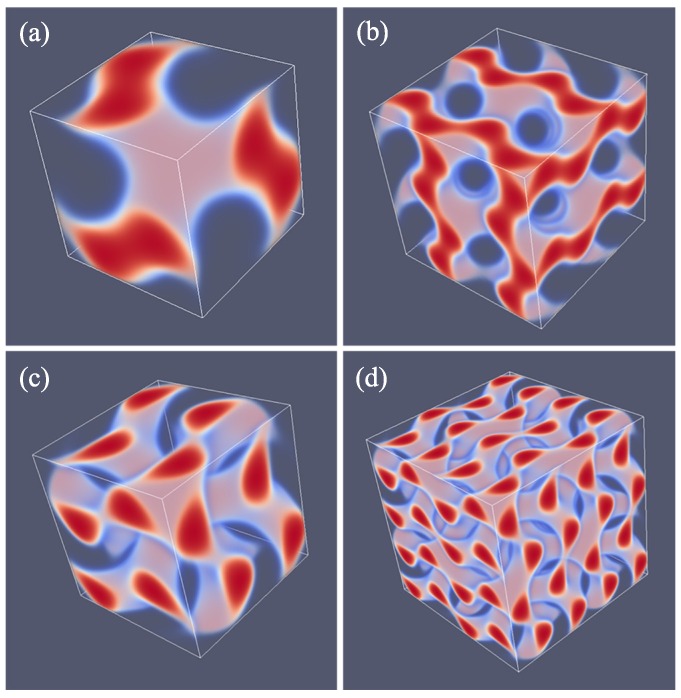
(**a**) SF-SCF predictions for the volume fraction profiles of a SG unit cell for D=43, N=300, χN=30 and f=0.33; (**b**) eight unit cells of a SG (result of (**a**) is doubled in each direction); (**c**) volume fraction profiles of a DG unit cell for D=74, N=300, χN=30 and f=0.34; (**d**) eight unit cells for the DG (result of (**c**) is doubled in each direction). The color coding is the same as in Figure 3.

Next, similarly as in other systems, we vary *L* systematically to find the optimal spacing. Here, we choose the spacing D=L for which the free energy density is minimized with respect to *L*. The SG phase has many interesting aspects that are well documented in the literature [21,22,23,24]. Our result for the SG phase closely follows all these features. As mentioned already, the SG lacks mirror symmetry. There only exists a three-fold symmetry axis along one of the diagonals of the unit cell as is easily seen from the profile given in Figure 4a. Other various features of SG appear when we multiply the unit cell a few times. As the majority phase is made transparent, we notice spherical holes with a hexagonal pattern when viewed from the view direction given in Figure 4b. From other view directions (not shown), one can see the holes in a square packing.

As proven below, the SG is metastable as one can easily find for f=0.33 either a HEX phase or a LAM phase for which the free energy density is lower. The reason for this relatively high free energy density is clear. The majority phase fills up 2/3 of the volume, whereas the minority phase fills the remainder of the volume in a gyroid-like. The interface in between the A and B rich domains deviates from the being a minimal surface (zero mean curvature throughout the interface), which is only expected for the SG gyroid phase with f=0.5. Such balanced SG phase does not exist as for f=0.5 the lamellar phase is the lowest in free energy.

A typical unit cell of a DG phase is depicted in Figure 4c. The viewgraph of eight unit cells (Figure 4d) now lacks the ‘holes’ because these holes are now the place in which the second gyroidal labyrinth of the minority phase is placed. Below, we show that the DG phase is a candidate ground state as it is lower in free energy than the HEX and the LAM for a small region of *f* values.

As compared to the SG, the DG has two gyroidal labyrinths with equal volume and the majority phase is in between the two and in this sense the symmetry is restored. From the perspective of the majority phase, the two sub-phases of the minority phase are spaced symmetrically around it even though f=0.33. As compared to the SG, the optimal spacing of the DG increased almost by a factor of two as it went up from D=43 for SG to D=74 for DG. Again, the DG has no symmetry planes and only one three-fold symmetry axis. Interestingly, the two minority phases only have three-fold junction points where three tubular domains come together. This appears sufficient to generate a so-called triply periodic structure, i.e., one can travel inside a minority phase in any of the three directions, *x*, *y* and *z* throughout the system.

**Figure 5:**
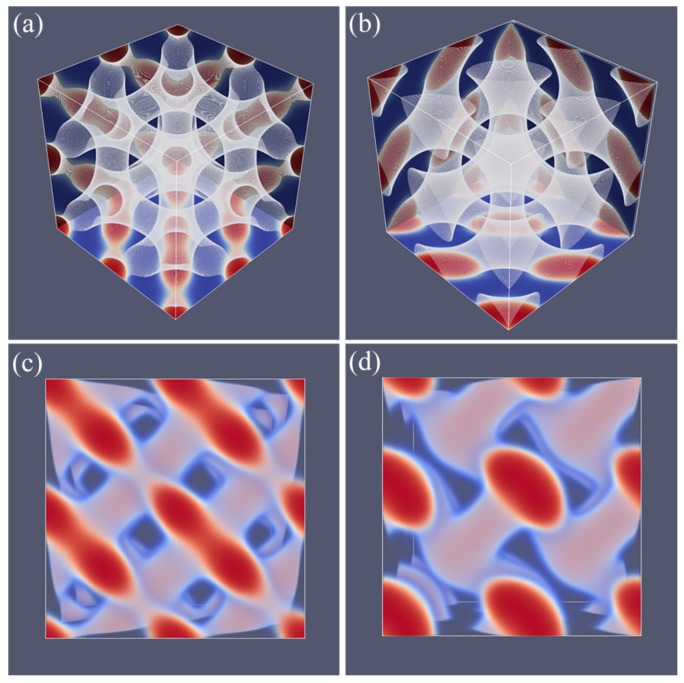
(**a**,**c**) Three-gradient equal density contour plots for eight unit cells of the double diamond DD phase (**b**,**d**) three-gradient equal density contour plots for eight unit cells of the hexagonal perforated lamellae HPL phase. For (**a**,**b**), the two phases are given in the orientation that reveals the three three-fold symmetry planes. In panels (**c**,**d**), we present a side view of eight unit cells (viewgraphs with x−y or y−z or x−z planes perpendicular to the viewing direction are all similar). The color coding is similar to the one given in Figure 3. Parameters for DD phase: D=45, N=300, χN=30 and f=0.33, HPL phase: D=43, N=300, χN=30 and f=0.30.

As explained above, we do not impose a particular symmetry for a particular segregated state during the SF-SCF optimization procedure, but instead implement an initial guess and apply appropriate boundary conditions. By accident, one of the calculations targeted for a SG phase deferred to a HPL phase. A possible reason for this unforeseen result was found posteriori by the observation that the HPL is competitive free-energy wise with the SG for the *f* value that was selected in the computations (see also below). Upon inspection of the HPL phase, a strong resemblance with the DD was found and therefore we present these phases side by side in Figure 5. For both phases, we employed periodic boundary conditions, and again for both phases, we have systematically varied the box size *L* and present the resulting structure for the optimal spacing *D*.

Referring to Figure 5a,c we present the DD morphology from two perspectives: (a) a view from along one of the diagonals from which the three symmetry planes can be seen and (c) a view with any of the planes of the unit cell placed perpendicular to the viewing direction. The DD morphology has often been described in the literature [36,37] and the phase that was predicted by SF-SCF is completely in line with this. The structure has three symmetry planes that are rotated with respect to each other by 60${}^{\circ }$. Similarly as for the DG, the minority phase forms two non-intersecting triply periodic regions with opposite handedness, while the majority phase is draped in between the minority labyrinths. The morphology of DD (space group $Pn\bar{3}m$) is characterized by four-fold junctions similarly as in the tetragonal C-network in diamond (or the structure of methane).

In Figure 5b,d, we show the typical HPL phase with an optimized spacing for an asymmetry fraction of f=0.30 for which the free energy density is lower than that of the corresponding SG (see below). The two viewgraphs show the structure from two viewpoints identical to the ones given for DD: (b) gives the view in the direction along the axis of the unit cell. For this point, the three symmetry-planes can be seen, and (d) gives the view face-on with one of the planes of the unit cell perpendicular to the view direction. The comparison between panels (a), and (b) as well as (c) and (d) in Figure 5 are apparent and informative. Comparing HPL and DD, we notice that the curvatures of the interfaces are clearly similar. There are, however, important differences. As the DD has four-fold connections, in the HPL phase, there are only three-fold connections (as in gyroids). (This is not easily recognized from the viewgraphs, but this is better observed by inspection of the HPL from many viewing directions). As compared to the DD, the fourth connection is ‘broken’. As a consequence, the HPL phase is lamellar and not triply-periodic: one can peel off one layer after another layer from the HPL phase, which is best seen from the images of (b) and (d) in Figure 11. (b) and (d) correspond to the morphology when we have the compositions of f=0.30 and 0.50, respectively. The layers together feature holes in hexagonally organized patterns. In (b) at low *f*, the hexagonally ordered pores are more apparent because the lower volume of the minority phase does not alter the visibility of the holes. In between the holes, lamellae strongly bend toward a neighboring layer in the direction of the missing connector. Two neighboring lamellae have opposite bending characteristics; hence, there are two types of layers in the HPL phase which alternate, each having inversed curvatures. These two types of layers resemble the two networks with opposite handedness in DG or DD phases. Characteristic for the HPL phase is that the odd layers almost touch each other across the pores of the even layers and vice versa. Indeed, if at these places, the odd layers would re-establish their connection and when similar connections were made between the even layers, we restore exactly the DD phase without the need to modify the curvatures of the interfaces much (as is best seen from close inspection of the differences between Figure 5c,d). The reason for the HPL phase to have lamellae with locally strong curves is to ensure a homogeneous distance between the lamellae in the presence of pores in the lamellae. Such homogeneity in interlamellar distances is a desired property as this ensures homogeneous stretching of the copolymer blocks.

### 2.5. Systematic Dependences

**Figure 6:**
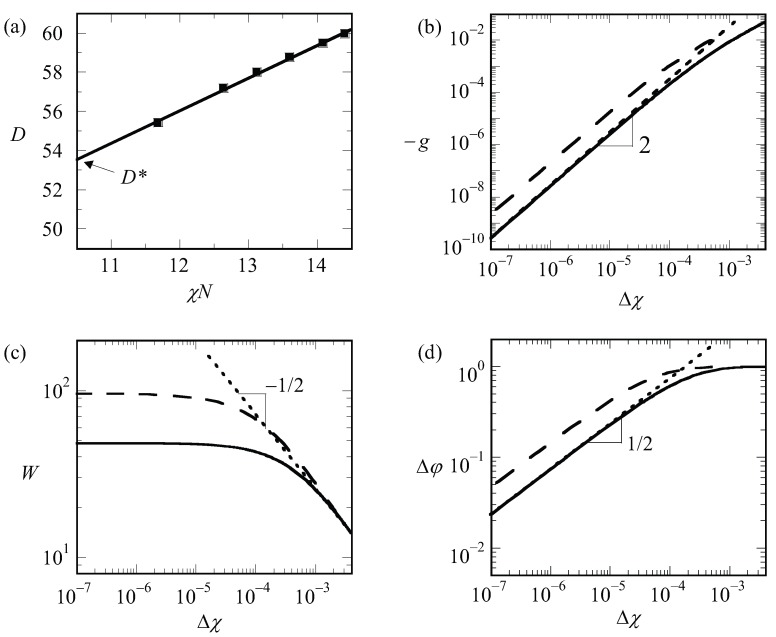
(**a**) The optimal spacing *D* in lattice units b plotted as a function for χN and N=1600. Here, optimal spacing at the critical point $D^{*}=D\left(10.5\right)$ is obtained by the linear fit; (**b**) absolute value of free energy density (−g) (in units of $k_{B}T/b^{3}$) as a function of Δχ in double logarithmic coordinates; (**c**) the width of the interface (in units **b**) as a function of Δχ in double logarithmic coordinates; (**d**) density difference (Δφ) as a function of Δχ in double logarithmic coordinates. $D^{*}/2$ values of 76 and 150 were used for *N* = 12,996 (solid lines) and 50,624 (dashed lines), respectively, in plots (**b**–**d**). Dotted lines represent the fits. The slopes are indicated on the plots.

By examining finite chain length effects near the critical region, we are faced with a problem. In order to find $\chi^{cr}$, we need to have an accurate guess for the optimal spacing at the critical point, which we will refer to as $D^{*}$. The latter we can only find by extrapolation. The (approximate) procedure that we followed is illustrated by referring to Figure 6a. In this graph, we present the optimal spacing of the lamellar phase with f=0.5 for a given overall chain length of N=1600 as a function of the product χN. Hence, for this graph, we varied the χ only and each point on the graph is the result from an optimization of the box size. We find $D^{*}$ by extrapolation to χN=10.5. It would have been better if we would have extrapolated to the exact critical value for the chain length N=1600 (which we will find below slightly lower than 10.5); however, the error in $D^{*}$ that is introduced in this way is negligible. We can collect $D^{*}$ as a function of chain length but which is presented later. We first analyze how the LAM phase is changed when we approach the critical point. These results are shown in Figure 6b–d, wherein we present the free energy density, the width of the interface and the density difference, respectively, as a function of $\Delta \chi =\chi -\chi^{cr}$ for a lamellar phase with f=0.5 for which $D^{*}$ is very close to an integer number. Hence, for such a case, we know that in the limit of Δχ→0, we enforce an optimal spacing to the system. For systems away from the critical region, one possibly would have acquired slightly different *D* values, but this requirement cannot be implemented in a lattice model.

As can be seen from Figure 6b,d, we obtain power-law dependences for both −g(Δχ) as well as Δφ(Δχ). Indeed, these scaling dependences have been employed to identify $\chi^{cr}$: the value of $\chi^{cr}$ was adjusted until the best power-law scaling was observed in Figure 6b,d in the limit of Δχ→0. Inspection of Figure 6b shows that we find $-g\propto \Delta \chi^{2}$. This value should be contrasted to the well-known result for the interfacial tension mentioned above for the liquid–liquid interface for macroscopic phase separation for which the coefficient is 3/2. We attribute the increase in the exponent to the observation that the width of the interface does not diverge (see Figure 6c). At high value of Δχ, the free energy density −g tend to level off a bit as it becomes more linearly dependent on Δχ.

Inspection of Figure 6d shows that $\Delta \varphi \propto \Delta \chi^{1/2}$ in the limit of Δχ→0 and goes to the constant value of unity for large values of the interaction parameter. The scaling exponent near critical is identical to the one found for macrophase segregation.

The width of the interface has a more complex behavior as illustrated in Figure 6c. It was already mentioned above that the width of the interface cannot exceed $D^{*}/2$. Hence, the width should level off in the limit of Δχ→0. In this case, the solid line is for N≈ 13,000 while the dashed line is for N≈ 50,000. The corresponding values for $D^{*}/2$ are 76 and 150, respectively. Upon inspection of the limiting value of the width, we notice that the width goes to approximately 2/3 of the value of $D^{*}/2$. Interestingly, for rather high values of Δχ, the width of the interface follows approximately $W\propto \Delta \chi^{-1/2}$, similar to that in the macrophase segregation problem. Apparently as long as $W\ll D^{*}/2$, we witness an increase of the interfacial width as if the two blocks would have been disconnected.

**Figure 7:**
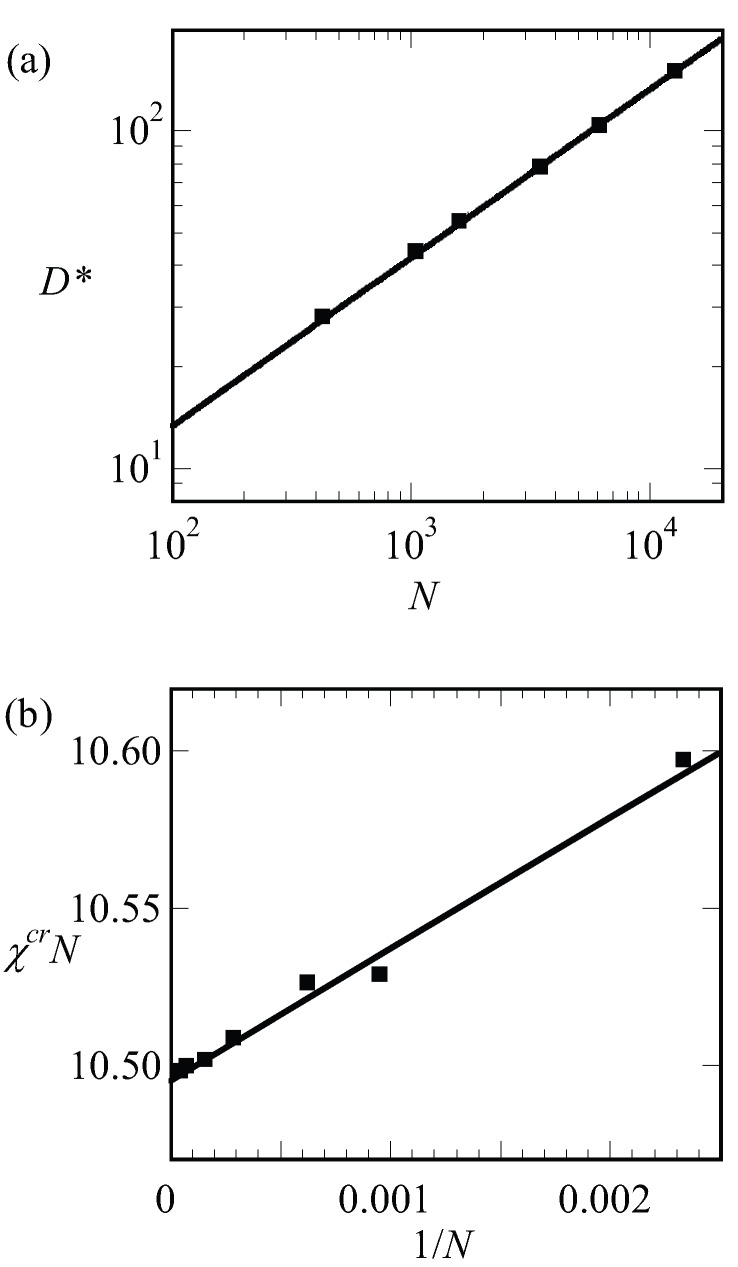
(**a**) The spacing at the critical point $D^{*}$ of a lamellar phase at f=0.5 as a function of the chain length *N* in double logarithmic coordinates. The fit of $D^{*}=\frac{4}{3}\sqrt{N}$ is drawn to guide the eye; (**b**) *N*-dependence on the critical χN value. The function $\chi^{cr}N=10.495\left(1+\frac{4}{N}\right)$ is drawn to guide the eye.

We have collected the optimal spacing at the critical point $D^{*}$ for a wide range of chain lengths and present these results in Figure 7a. To a good approximation, the results are represented by $D^{*}=\frac{4}{3}\sqrt{N}$, which is consistent with early predictions of Leibler [15]. Next, we collected the critical interaction parameter $\chi^{cr}$ again for a wide range of chain lengths and present the results in Figure 7b. In this graph, we have plotted $\chi^{cr}N$ as a function of 1/N and found to a good approximation as a straight line. The fitting result is indicated in the legend of Figure 7b. Within the accuracy of the fitting procedure, we thus find $\chi^{cr}N=10.495\left(1+\frac{4}{N}\right)$. These results are compatible with finite chain length corrections for the critical point found in the literature [17].

**Figure 8:**
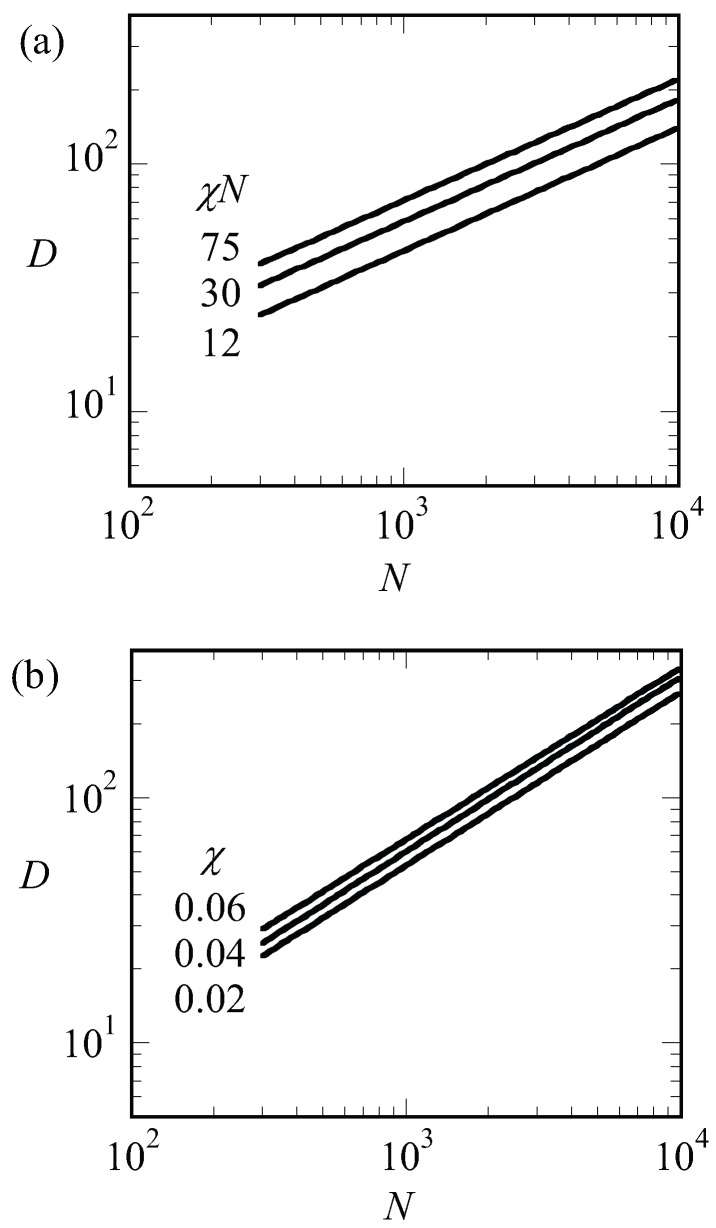
(**a**) The optimal spacing (*D*) as a function of the chain length (*N*) in lamellar phase for χN values labeled next to the lines in double logarithmic coordinates. All lines have a slope of 1/2; (**b**) the optimal spacing (*D*) as a function of the chain length (*N*) in lamellar phase for fixed χ values that are labeled next to the lines in double logarithmic coordinates. All lines have a slope of 2/3.

We may use a simple Flory-like argument to elaborate on the scaling of the spacing with the chain length. In this argument, we can balance the entropic penalty for stretching of the chains, written as $D^{2}/N$ (ignoring numerical coefficients) with the free energy to enlarge the contact area between the A and B blocks, γa, where γ is the interfacial free energy associated with this surface and *a* is the area per molecule. The latter can be estimated from the filling of the system by chains, hence a=N/D and thus the free energy per chain is

(7) $$F=\frac{D^{2}}{N}+\frac{\gamma N}{D},$$

where the free energy per chain is in units of $k_{B}T$ and that numerical coefficients are ignored. Optimization with respect to *D* gives $D=\gamma^{1/3}N^{2/3}$. The interfacial energy of an A–B interface is known to depend on the square root of the interaction energy, $\gamma \propto \chi^{1/2}$ [42]. Using the result that at the critical point $\chi \propto N^{-1}$, we notice that $D\propto \gamma^{1/3}N^{2/3}=N^{-1/6}N^{2/3}=\sqrt{N}$. We can apply the same argument to systems that are not close to the critical point. In Figure 7a, we present the optimal spacing for LAM phase with f=1/2 for low, intermediate and strong segregation regions of χN=12,30 and 75, respectively, as a function of the chain length *N*. Hence for each of the lines, we varied χ such to keep χN fixed. Again, we find that the spacings obeyed $D\propto \sqrt{N}$ to a good approximation. This must be contrasted to the results presented in Figure 8b. In this figure, we present the optimal spacing for the symmetric LAM phase (f=1/2) as a function of the chain length for fixed value of the interaction parameter. Hence, in these cases, we only changed *N* and the interfacial energy γ was approximately constant. As a result, the spacings obeyed $D\propto N^{2/3}$ to a good approximation.

**Figure 9:**
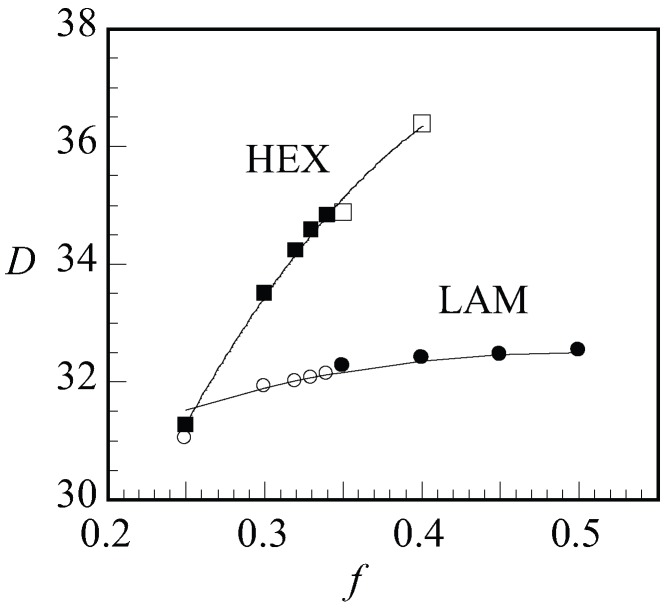
Optimal spacing (*D*) of LAM and HEX phases as a function of composition (*f*) for N=300 and χN=30. For LAM phase, the fit of $D\propto f^{2/3}+{\left(1-f\right)}^{2/3}$ is drawn to guide the eye. Open and closed symbols refer to the metastable and stable points, respectively.

Next, we present the optimal spacing *D* as a function of *f* for LAM and HEX in Figure 9 for a given value of N=300 and χN=30. When changing *f*, we will pass the phase boundary around f=0.34. Hence, in Figure 9, we showed the data points of the stable phases as filled dots and squares for LAM and HEX, respectively, and data points for the non-stable phases were shown as empty. With increasing *f*, we find an increase in the optimal spacing. For the LAM phase, the slight increase in the spacing to a plateau region was successfully fitted by $D\propto f^{2/3}+{\left(1-f\right)}^{2/3}$. This is the expected dependence when the two blocks independently find the optimal width. A significantly stronger dependence for D(f) was found for the HEX phase where to a reasonable approximation $\propto f^{1/3}$. This result must be attributed to the decreased space occupied by the minority block in the cylindrical domains.

We would like to report on the chain length dependence of the HEX to LAM phase transition $\left(f^{tr}\right)$ at a finite value of χN. We tried to obtain such result for χN=30. We tested a range of chain lengths of N=100−1000. Within the numerical noise of our procedure, we could not detect the expected chain length dependence for $f^{tr}$. This may be attributed to the noise in the data, which was amplified slightly because we could not satisfy the box dimensions for the HEX phase $L_{x}/L_{y}=\sqrt{3}$ with enough precision. Instead, we focused on the stability of the various mesophases in the neighborhood of $f^{tr}$.

**Figure 10:**
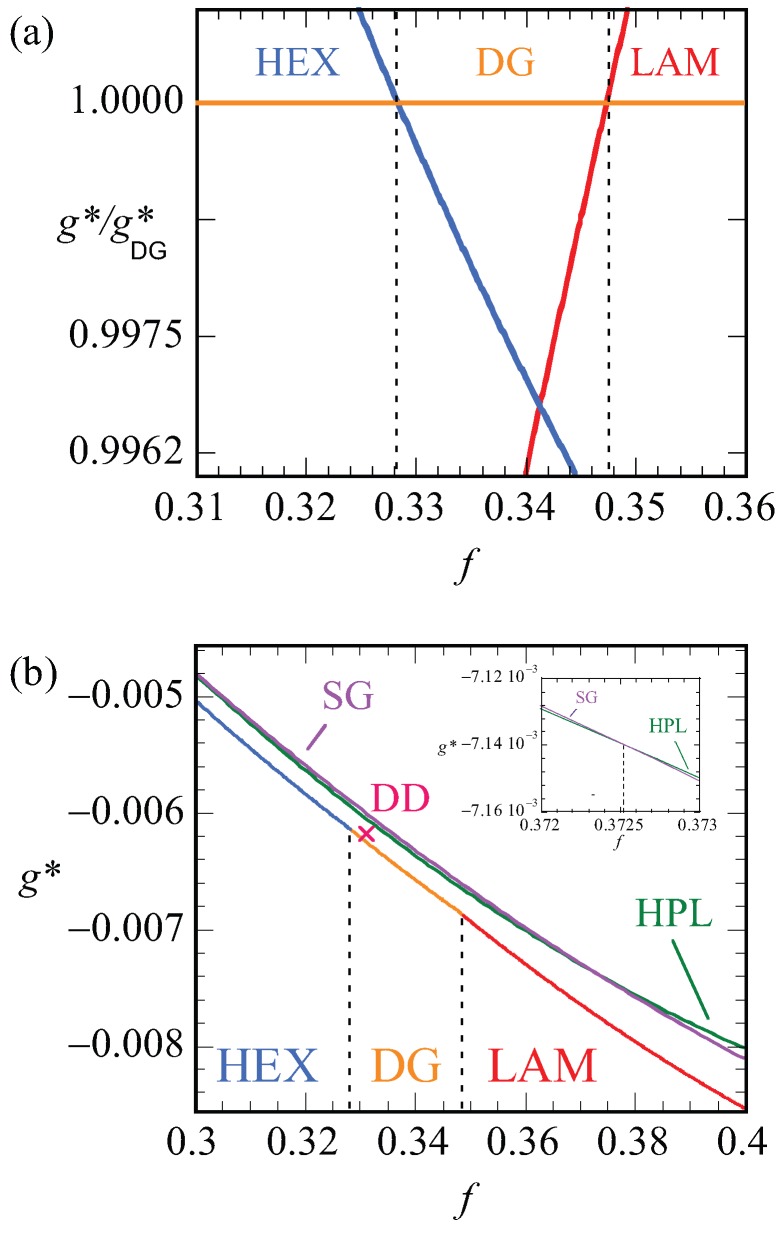
(**a**) Optimized free energy density $g^{*}$ in units of $k_{B}T$ of HEX (blue) and LAM (red) phases divided by the free energy density of DG ($g^{*}/g_{DG}^{*}$) as a function of composition *f* for N=300, χN=30. Stable phases of HEX and LAM occur when their $g^{*}/g_{DG}^{*}>1$. First, second and third regions, separated by dotted lines, represent the HEX, DG and LAM stable regions; (**b**) free energy density ($g^{*}$) of HEX, LAM, DG, DD, SG and HPL phases as a function of composition (*f*) for N=300, χN=30. DD phase is shown in one point only. Only stable regions of HEX, DG and LAM are shown in the graph. The inset gives an expanded view near the crossing point of the HPL and SG free energy densities as a function of *f*.

Let us focus next on the stability of various mesophases near $f^{tr}$. For all phases, we have computed the optimized free energy density as a function of the asymmetry ratio, $g^{*}\left(f\right)$. Plotting these free energy densities as a function of the asymmetry fractions results in lines that are nearly parallel (cf. Figure 10b). The results are more easily discussed when we take the ratio $g^{*}/g_{DG}^{*}$ as a function of *f*. Obviously, this dependence results in unity for the DG phase. Recalling that the free energy densities are negative, we find that when the ratio is smaller than unity, i.e., when $g^{*}<g_{DG}^{*}$ the DG is preferred and when $g^{*}>g_{DG}^{*}$ the other phase is stable. In Figure 10a, we show this ratio as a function of *f* for the HEX, the LAM and the DG phases. Literature points to a stability domain for the DG phase between f=0.320−0.349 [6]. We found DG as the most stable phase between f=0.328−0.347. These results are consistent with each other also considering the fact that the SF-SCF results are for a fixed chain length N=300.

In Figure 10b, the bottom line presents the lowest free energy values that were obtained, i.e., it connects the data of the HEX phase to those of the DG and then to the LAM phase. The vertical dashed lines present the phase boundaries. The point X gives the free energy density of the DD phase, which is indeed slightly larger than the DG at the same *f* value. The two top lines are the free energy densities of the HPL and the SG phases. Both these phases are metastable as their free energy density is higher than the HEX, DG, LAM phase boundary. As shown in the inset, the free energy densities of the HPL and SG cross each other around f=0.37. For smaller *f* values, the HPL is preferred over SG and, above this crossing point, the SG is more stable.

**Figure 11:**
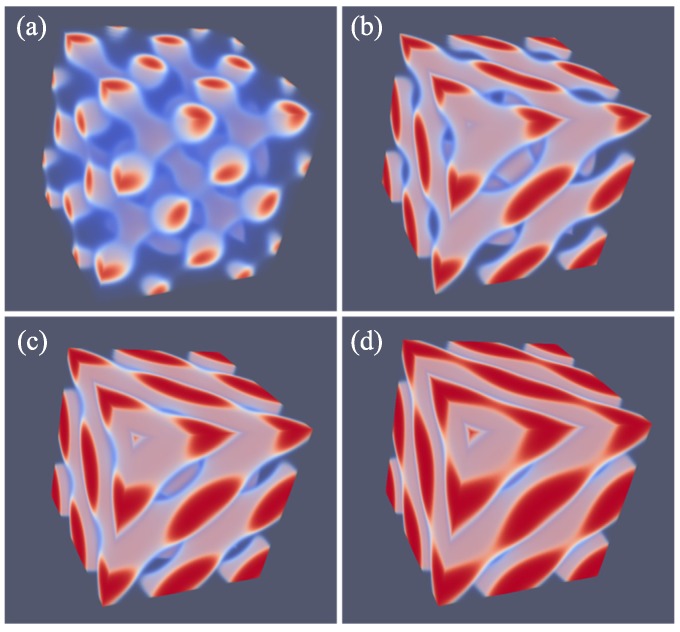
Equal density contour plots for the HPL phase for N=300, L=43 and χN=30. (**a**–**d**) correspond to *f* values of 0.19, 0.30, 0.37 and 0.50, respectively. Unit cells were doubled in each direction. Color coding as in Figure 3. Note that we did not optimize the box size in this case.

Of all phases that we have considered, the HPL phase is possibly the most novel one. It turned out that the phase is computationally highly robust and it was quite easy to find HPL phases for various values of *f* covering the whole relevant range from the region where the LAM phase is stable (e.g., f=0.5) to near the order–disorder line for f<0.2. In Figure 11, we put four variants of the HPL phase for f=0.19, 0.30, 0.37 and 0.50 side by side. The first one is the HPL in the region where spherical phase is the preferred state; (b) is near the region where the HEX is the stable phase; (c) is a transition state between HEX and LAM, e.g., DG; (d) is the HPL for the case where obviously the LAM phase is the ground state. It is noticed that at large values of *f*, the lamellae of the HPL are close to planar (f=0.50, i.e., (d)) and the hexagonally ordered holes are relatively small. The holes become bigger when *f* decreases. At the same time, the layers become more strongly curved (f=0.30, i.e., (b)). Now, the connection points are more apparent as three cylindrical domains come together. When *f* is decreased even more (f=0.19, i.e., (a)), the cylindrical domains are no longer homogeneous in thickness. Instead, the tubular fragments develop similarly to spherical regions with a neck in between. This is consistent with spherical detached domains being preferred for systems with such low *f* values. For small *f* values, the minority phase becomes smaller and smaller in volume and therefore the holes where the majority phase exists in the HPL phase grow larger and larger. In other words, the tubular regions that keep the layers intact become thinner and thinner. The lamellae also become more curved and the direction of the thin tubular fragments are almost perpendicular to the planar lamellae found close to f=0.50.

**Figure 12:**
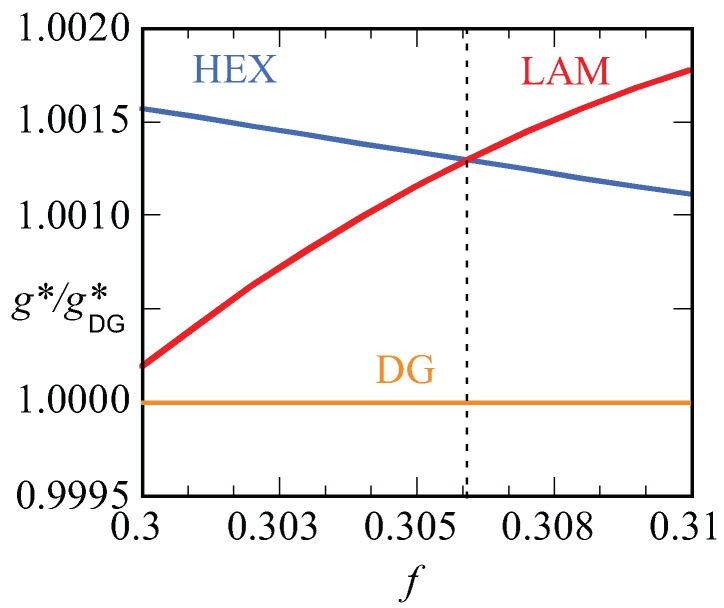
Free energy density of HEX (blue) and LAM (red) phases divided by the free energy density of DG (orange) $\left(g^{*}/g_{DG}^{*}\right)$ as a function of composition *f* for the system N=300, χN=120. Stable phase occurs when the corresponding phase has the value of $g^{*}/g_{DG}^{*}>1$. HEX and LAM phases are stable in left and right regions, respectively, that are separated with a dotted line.

In Figure 12, we present our results for the strong segregation, χN=120. We kept the chain length at N=300, and thus increased the interaction parameter. Here, we plot the free energy density $g^{*}$ normalized by this value for the DG (similarly as in Figure 10a) as a function of the *f* value. Again, only when this ratio is smaller than unity do we expect the DG to be stable (recall that the free energy density is negative). Inspection of Figure 12 proves that the ratios are larger than unity for both the HEX as well as the LAM phase. This proves that the DG phase is metastable for these high values of the interaction parameter. The free energy densities of the HEX and LAM phases cross at f≈0.3061, which is below the value found for the HEX-LAM transition region for χN=30. This is in line with the general knowledge of the phase diagram that the phase transitions all move slightly to smaller *f* values with increasing χN. We note that the ratio $\frac{g^{*}}{g_{DG}^{*}}$ are just of order 1.0015, which means that the free energy differences are minor. Nevertheless, the differences are significantly larger than the noise in the data (data point not shown are exactly on the lines), and we believe that these results are sufficiently accurate.

A typical value for the optimal *D* for the DG at χN=30, N=300 was D=74. The optimal spacing for the DG at χN=120 for N=300 near f=0.31 was found to be D=101. This growth with a factor 1.36 in optimal spacing is a bit larger than expected from $D\propto \gamma^{1/3}N^{2/3}$, which implies $D\propto \chi^{1/6}$ and hence a growth by only a factor 1.26. We also investigated the free energy density of the HPL phase for the strong segregation χN=120. In line with the results at lower segregation, the HPL phase is also metastable and indeed the DG phase outperforms the HPL phase also at χN=120 as $\frac{g_{HPL}^{*}}{g_{DG}^{*}}=0.991$ (the DG free energy density $g^{*}\approx -0.583$ while that of the HPL is $g^{*}\approx -0.578$ for the corresponding *f* values).

## 3. Discussion

Microphase segregation of block copolymers is a rich topic. Experimentally, there are many challenges when it comes to exploit all the potential features of these systems to the best. This is why theoretical investigations are still timely. We forwarded the results of SF-SCF calculations for finite chain lengths and complement existing modeling efforts [6,7]. Indeed, most of the predictions mentioned above are fully in line with the common knowledge of microphase segregation. It is not necessary to re-iterate all of these, but a few of these must be mentioned once again. Most prominently, we showed finite chain length corrections near the critical region are of order 1/N. These corrections have been established before and it shows that the SF-SCF method is accurate and reliable.

The second aspect of microphase segregation that we focused upon was the stability of the DG phase. We found that at intermediate segregation χN=30 for N=300, and the DG phase has a narrow region of stability in between the HEX and LAM phase. For fixed *N*, we showed that a fourfold increase in χ made the stability window disappear. The later result is not in line with recent SCF calculations, which suggested that at high χN the DG should remain stable [7]. We must also mention that the high χN limit of 120 can be reached in two ways. Either the chain length is increased at fixed value of the interaction parameter, or the interaction parameter is increased at fixed chain length. We have chosen the latter. It is also not excluded that when the chain length would have been increased at fixed χ, the outcome could have been different because then the interfacial width is preserved. Nevertheless, by increasing χ, the width of the interfaces decreases and this may have destabilized the DG phase compared to the HEX or LAM phases, which have curvature-wise smoother surfaces.

We understand that the problem of stability of the DG phase at strong segregation is not solved by our finding yet. An extra argument could possibly be found by computing the Gaussian bending modulus of the corresponding interfaces [2]. When this parameter is positive, we may expect the appearance of phases with saddle shapes (e.g., as in the DG phase), but when it is negative we do not expect such phases to be stable. Unfortunately, we do not know how to compute the Gaussian bending modulus in microphase segregation systems directly. When our results are accurate, we expect that the Gaussian bending modulus is negative far from the critical point (strong segregation, there is no DG phase) and may be positive near the critical point (DG or phases that have saddles are stable). We do know how to compute the bending moduli of membranes and liquid–liquid interfaces [12,13,14] and we can apply this to cases where there are surfactants (copolymers) at liquid–liquid interfaces (microemulsions). We have preliminary (unpublished) results from which it appears that the Gaussian bending modulus is positive near the critical point of such systems, but negative far from such critical point. By addition of a pair of solvents (one for each phase), we can transform the microphase segregation system to a microemulsion system [43]. Hence, it is not far fetched to extrapolate the finding in microemulsions to the microphase segregation system. If such extrapolation is valid, we may find extra arguments for our prediction. We thus do not exclude that additional evidence for the disappearance of the DG phase at strong segregation might in due time come from a systematic study of the (sign of the) Gaussian bending modulus.

We may also speculate that the metastability of the DG phase at high χ values is due to lattice artifacts. Indeed, when the interfaces sharpen it to such an extent that the width is of the interface is not wide compared to the discretization, the path followed by the interface may deviate from the best possible path. In other words, the interfaces follow low energy paths provided by the lattice rather than the ones dictated by stretching, area minimization and volume filling. As a result, one would overestimate the free energy density of the phases that cannot optimize the position of the interface with respect to the lattice. We believe that this is not what caused the metastability of the DG to occur in our case, but, in order to exclude this option, we need to implement a lattice refinement study.

There are several interesting aspects of the SF-SCF approach for further studying copolymer microphase segregation. There are a number of modifications of the system, which are readily available in the existing implementations of the theory, that allow one to undertake similar studies for: (i) multiblock copolymers; (ii) polydisperse copolymers [44]; (iii) additions of solvents with non-trivial partitioning; (iv) branched or topologically complex chain architectures [45,46]; including (v) longer ranged interactions, for example electrostatic interactions [47,48,49].

In the above, we have focused on the stability issue of DG phase, which occurs near the HEX to LAM transition. We believe that the region between spherical and HEX phases is also of interest for further investigations. We have preliminary data showing that a phase of short dumbbells is stable at high value of χN. Such phases may be isotropic (dumbbells oriented in three), possibly in six directions e.g., *x*-, *y*- and *z*-directions as well as in three diagonal directions but also anisotropic (dumbbells oriented all parallel or ordered in two directions e.g., in planes). Such phases may have interesting optical properties when the refractive index of the minority phase is sufficiently different from the majority phase. Work in this direction is in progress. Plus, a unified work involving the comparison of experimental and modeling results obtained using the SF-SCF method can play an important role for the researchers to produce these attractive block copolymer nanostructures with a high precision [6,34].

Finally, we may reflect on our HPL results. There are several reports in the literature about HPL phases in SCF modeling [50,51]. We are not absolutely sure whether we have found the same HPL as reported previously, and that is why we extensively presented the density profiles. We have seen in the calculations that our HPL phase is highly robust. We may speculate that this also has a physical interpretation. It might explain why, in experimental cases, the HPL phase is relatively frequently reported as being metastable after annealing block copolymer films [34,35]. The HPL phase has the features close to the disconnected spheres when f<0.2, it shows features of the hexagonal cylinders at intermediate f≈0.3 and it is close to lamellar when f≈0.5. We then take this result to speculate that the HPL phase is an intermediate phase that ‘transports’ the system from one phase into the other. We have seen how it resembles, e.g., the DD when f=0.3, we see how it can transform into lamellae when the *f* value is larger, or how it can transform the system into a hexagonal phase when the *f* value is smaller. Such transitions may be possible upon the addition of a selective solvent that effectively changes the *f* value in the system. Such insights may help experimentalists to further explore the microphase segregation phenomenon.

## 4. Conclusions

We have used the SF-SCF formalism to study microphase segregation of block copolymers. Most of our results are in line with previously undertaken SCF studies. We contributed to the problem by focusing on finite chain length effects. We believe that the SF-SCF method is appropriate to investigate microphase segregation of block copolymers and remains accurate at strong segregation because of the non-local contributions in the segment potentials being considered. We analyzed the structure of the LAM phase in the neighborhood of the critical point and reproduced the finite chain length corrections near the critical point. We found that the free energy density and the density difference scales with the distance to the critical point as a power-law. The width on the interface remains finite. We found that at intermediate segregation the DG phase has a narrow stability region in between the HEX and LAM phases and, most significantly, we predicted that, for strong segregation, that is, for high χ-values, this stability domain vanishes. We note that the computation strategy is unbiased with respect to the symmetry of the solution. We only need an initial guess and appropriate boundary conditions to focus on a particular solution. The rich structure of the HPL is an example of the type of results that one obtains by the SF-SCF method. The curvature of the lamellae as well as the size of the holes and the undulations in the connectors all vary with the asymmetry ratio *f*, and these effects made us speculate about the role of HPL to mediate the system from one phase into another.

## Appendix A. Details of the Scheutjens–Fleer Self-Consistent Field Method

Let us consider lattice sites with length of *b* and volume of $v=b^{3}$. Below, all lattice lengths are given in units of *b*, which are approximately 0.5 nm in real size. Space is built up by a simple cubic ordering of these sites. Introducing a Cartesian coordinate system of lattice sites, $x=1,2,\cdots ,L_{x}$, $y=1,2,\cdots ,L_{y}$ and $z=1,\;2,\;\cdots ,\;L_{z}$. We will refer to a particular site by r=(x,y,z). The dimensionless volume of the system (box) is given by $V=L_{x}L_{y}L_{z}$. The idea of the specified volume is that it will represent a unit cell of the system of interest. To properly implement this, we need boundary conditions. As these boundary conditions depend on the type of system that is aimed for, we will pay attention to this separately below.

In this system, we introduce flexible copolymer chains that are built up by a linear string of spherically symmetric segments such that each segment occupies one lattice site. Let the composition be given by $A_{N_{A}}B_{N_{B}}$ where segments $s=1,\;2,\cdots ,N_{A}$ are of type A and the remainder of the segments are of type B, i.e., for $s=N_{A}+1,\cdots ,N$, where the total number of segments per chain is given by $N=N_{A}+N_{B}$. Hence, the fraction of A segments is given by $f=N_{A}/\left(N_{A}+N_{B}\right)$. Below, we will adopt the freely jointed chain (FJC) model to describe the chain statistics. In this model, two consecutive segments along the chain occupy neighboring lattice sites, but longer ranged correlations are ignored.

Below, we will use a mean field approximation. In such an approach, the key quantity is the probability that a site is filled with segment type A or B and will be given by volume fractions of the $\phi_{A}$ and $\phi_{B}$, respectively. One way to envision such volume fractions is that we consider not just one box, but an ensemble of boxes and that the focus is on the ensemble average.

It is a good approximation to consider most liquids as incompressible. This also applies to copolymer melts. We therefore implement an incompressibility condition on each site. This means that we are only interested in the solution for which at each coordinate *r*, when $\varphi_{A}\left(r\right)+\varphi_{B}\left(r\right)=1$. It is convenient to define so-called site averages, e.g., for the segment density, which is denoted by angular brackets and encompasses an average of the volume fractions over the nearest neighbor coordinates:

(A1) $$\begin{array}{c} \langle \phi \left(x,y,z\right)\rangle =\frac{1}{6}[\phi \left(x-1,y,z\right)+\\ \phi (x+1,y,z)+\phi (x,y-1,z)+\\ \phi (x,y+1,z)+\phi (x,y,z-1)+\phi (x,y,z+1)]. \end{array}$$

It is known that, in the limit of small lattice spacings (weak gradients), the site average accounts for the local density as well as the curvature of the density in three directions:

(A2) $$\langle \varphi \left(r\right)\rangle =\varphi \left(r\right)+\frac{1}{6}\nabla^{2}\varphi \left(r\right).$$

In an exact lattice theory, the sites are filled either by a segment A or B and the local segment interactions should be evaluated accordingly to the instantaneous surroundings. Within mean field approximations, the exact counting of contacts is replaced by an estimated counting using probabilities that sites are filled, e.g., based on the volume fractions. This simplifies the evaluation of the partition function because it then boils down to the evaluation of the so-called single chain partition functions, q, wherefore the molecules feel the surroundings by potential fields. Hence, in the mean field theory, there are, complementary to the volume fractions, so called segment potentials $u_{A}\left(r\right)$ and $u_{B}\left(r\right)$, ‘felt’ by the A and B segments, respectively. It can be shown that the mean field free energy (*G*) in terms of the volume fraction and segment potentials can be written as below, which is specified in units of the thermal energy $k_{B}T$:

(A3) $$\begin{array}{c} G=-\ln\frac{q^{n}}{n!}-\sum_{r}\left[u_{A}\left(r\right)\varphi_{A}\left(r\right)+u_{B}\left(r\right)\varphi_{B}\left(r\right)\right]\\ +\sum_{r}\frac{1}{2}\chi \varphi_{A}\left(r\right)\langle \varphi_{B}\left(r\right)\rangle +\varphi_{B}\langle \varphi_{A}\left(r\right)\rangle ]\\ +\sum_{r}\alpha \left(r\right)\left[\varphi_{A}\left(r\right)+\varphi_{B}\left(r\right)-1\right]. \end{array}$$

Here, n=V/N is the number of copolymers in the volume. The factor 1/2 is present to correct for the double counting as the two terms within the square brackets are the same after summation over r has taken place. The incompressibility constraint is added to the mean field free energy though the use of a Lagrange parameter α(r). This will allow for an easier differentiation of the mean field free energy. It turns out that we need a saddle point of *G*; that is, we need a maximum of *G* with respect to the segment potentials and the Lagrange field and a minimum with respect to the volume fractions. The search for such a saddle point leads to the self-consistent field protocol, which specifies (i) the rule about how to compute the segment potentials from the volume fraction; (ii) the rule about how to compute the volume fractions from the potentials and finally (iii) how to find the value for the Lagrange field. These rules will be discussed in the following sections. After the free energy is optimized, we can simplify it and make sure that it is normalized such that, when the system turns homogeneous, the free energy vanishes. We will return to this below.

When we take the derivative of Equation (A4) with respect to the volume fractions and set the result to zero, we find an equation for the segment potential. The result for segment type A is

(A4) $$\frac{\partial G}{\partial \varphi_{A}\left(r\right)}=u_{A}\left(r\right)+\alpha \left(r\right)+\chi \langle \varphi_{B}\left(r\right)\rangle =0.$$

Note that, in the segment potentials, the interaction term includes curvature information through the angular brackets. Again, when the curvature in the volume fractions is minor, we find that $u_{A}\left(r\right)=\alpha \left(r\right)+\chi \phi_{B}\left(r\right)$, and this form of the potential is used classically in the field of block copolymer self-assembly. Typically, we will normalize the potentials such that, when the system is homogeneous, the potentials are zero; hence, in practice, we use:

(A5) $$u_{A}\left(r\right)=\alpha \left(r\right)+\chi \left(\langle \varphi_{B}\left(r\right)\rangle -\left(1-f\right)\right),$$

(A6) $$u_{B}\left(r\right)=\alpha \left(r\right)+\chi \left(\langle \varphi_{A}\left(r\right)\rangle -f\right).$$

The optimization of the mean field free energy with respect to the segment potentials leads to the rule about how to compute the volume fractions:

(A7) $$\frac{\partial G}{\partial u_{A}\left(r\right)}=-\frac{n\partial \ln q}{\partial u_{A}\left(r\right)}-\varphi_{A}\left(r\right)=0.$$

Hence, we need the evaluation of the molecular partition function *q*. It turns out that subsequent differentiation of the lnq is not needed because the propagator formalism gives, in addition to the value of *q*, the volume fractions.

It is convenient to introduce the Boltzmann weights $G_{A}\left(r\right)=\exp-u_{A}\left(r\right)$ and $G_{B}\left(r\right)=\exp-u_{B}\left(r\right)$. Next, we introduce so-called free segment distribution functions $G\left(r,s\right)=G_{A}\left(r\right)$ when segment $s=1,2,\cdots ,N_{A}$ is of type A and $G\left(r,s\right)=G_{B}\left(r\right),$ otherwise. These quantities are used to compute so-called end-point distribution functions G(r,s|1) or G(r,s|N) with the propagator equation:

(A8) G(r,s|1)=G(r,s)〈G(r,s−1|1)〉,

(A9) G(r,s|N)=G(r,s)〈G(r,s+1|N)〉,

which are started by realizing that, at the ends of the chain, we can use the free segment distributions:

(A10) G(r,1|1)=G(r,1),

(A11) G(r,N|N)=G(r,N).

In passing, we note that when we Taylor expand the free segment distribution function G(r,s)=1−u(r,s) and replace the angular brackets as in Equation (A4) with the local end-point distribution plus the curvature contribution, we can rewrite the right-hand side of Equations (A8) and (A9) and obtain four terms on the right-hand side. Neglecting the product of the potentials and the second derivative (both should be small), we can arrange the result and obtain the Edwards diffusion equation [52]:

(A12) $$\frac{\partial G}{\partial s}=\frac{1}{6}\nabla^{2}G-uG.$$

Note that the latter equation is applicable for Gaussian chains, whereas the propagators are representing FJCs. There is only a noticeable difference between the Gaussian chain and the FJC when the chains become strongly stretched in the limit of their contour length. This will not easily occur for block copolymer self-assembly. The partition function can now be computed by:

(A13) $$q=\sum_{r}G\left(r,N|1\right)=\sum_{r}G\left(r,1|N\right)$$

and the combination of two complementary end-point distribution functions leads to the segment densities:

(A14) $$\varphi \left(r,s\right)=\frac{n}{q}\frac{G(r,s|1)G(r,s|N)}{G(r,s).}$$

In this equation, the division by G(r,s) is needed to prevent that the statistical weight for the segment *s* is counted twice. Finally, the volume fractions of the segment types become:

(A15) $$\varphi_{A}\left(r\right)=\sum_{s=1}^{N_{A}}\varphi \left(r,s\right),$$

(A16) $$\varphi_{B}\left(r\right)=\sum_{s=N_{A}+1}^{N}\varphi \left(r,s\right).$$

Optimization of the free energy (Equation (3)) to the Lagrange parameter α(r) gives the compressibility constraint (Equation (1)). Below, we discuss the iteration scheme, but part of this is the need to know the Lagrange field. This quantity is updated at iteration *k* simply by adding the constraint:

(A17) $$\alpha {\left(r\right)}^{\left(k\right)}=\alpha {\left(r\right)}^{\left(k-1\right)}+\eta \left(\varphi_{A}{\left(r\right)}^{\left(k\right)}+\varphi_{B}{\left(r\right)}^{\left(k\right)}-1\right).$$

The idea behind Equation (A17) is the following. When the local volume fractions do not add up to unity, the value of the Lagrange field should be adjusted. When the overall volume fraction (density) exceeds unity, we need to push segments away from that coordinate. This is done by increasing α. Inversely, when the volume fraction is less than unity, we need to attract segments to this site. We do this by decreasing α. Equation (A17) will do this exactly. In Equation (A17), η<1 is a damping parameter. Typically, a value of 0.1 is needed to find smooth convergence.

The above set of equations is closed. As mentioned already, we need an iteration to find the fixed point. Let us define the interaction term, $E_{A}\left(r\right)=u_{A}\left(r\right)-\alpha \left(r\right)$ and $E_{B}\left(r\right)=u_{B}\left(r\right)-\alpha \left(r\right)$. According to Equation (5), we can compute this interaction term when the volume fractions are available. Let’s assume that, at iteration of k−1, we have the interaction terms $E_{A}{\left(r\right)}^{\left(k-1\right)}$ and $E_{B}{\left(r\right)}^{\left(k-1\right)}$ as well as the potentials $u_{A}{\left(r\right)}^{\left(k-1\right)}$ and $u_{B}{\left(r\right)}^{\left(k-1\right)}$. The latter ones are used to compute volume fractions (cf. Equation (A12)) $\varphi_{A}{\left(r\right)}^{k}$ and $\varphi_{B}{\left(r\right)}^{k}$. With these, we may evaluate $E_{A}{\left(r\right)}^{k}$ and $E_{B}{\left(r\right)}^{k}$. Next, we use Equation (A17) to find an update for the Lagrange field $\alpha {\left(r\right)}^{k}$ and update the segment potentials, e.g., for segment type A, according to the Pikar mixing:

(A18) $$u_{A}{\left(r\right)}^{k}=\alpha {\left(r\right)}^{k}+\eta E_{A}{\left(r\right)}^{k}+\left(1-\eta \right)E_{A}{\left(r\right)}^{(k-1).}$$

Now, the loop is closed and we can continue until the potential fields as well as the segment volume fractions between two consecutive iterations differ less than a tolerance value. We typically continue until we have seven significant digits. Note that this Pikar-like iteration does not require the storage of a large matrix and hence can be used also for three-gradient applications.

In practice, we do not use this Pikar scheme because it needs relatively many iterations. Instead, we use target functions specified by Evers et al. [53] and use the direct inversion in the iterative subspace (DIIS) algorithm [41] to find the fixed point. The method is remarkably fast and typically requires only an order of 100 iterations, and, even for 3-gradient applications, one obtains solutions within a few minutes of CPU time. When necessary, the procedure can be implemented on graphical cards, which, for large enough systems, can lead to improvements in wall time by a factor of 5 to 10.
